# A systematic review and meta-analysis of the clinical performance of implant-supported overdentures retained by CAD-CAM bars^*^

**DOI:** 10.1590/1678-7757-2023-0054

**Published:** 2023-08-25

**Authors:** Ana Paula Chappuis-Chocano, Helena Sandrini Venante, Rodrigo Moreira Bringel da Costa, Mariana Domingues Pordeus, Oscar Oswaldo Marcillo-Toala, Joel Ferreira Santiago, Vinícius Carvalho Porto

**Affiliations:** 1 Universidade de São Paulo Faculdade de Odontologia de Bauru Bauru São Paulo Brasil Universidade de São Paulo, Faculdade de Odontologia de Bauru, Bauru, São Paulo, Brasil.; 2 Universidad de Especialidades Espíritu Santo Samborondón Ecuador Universidad de Especialidades Espíritu Santo (UEES), Samborondón, Ecuador.

**Keywords:** Denture, Overlay, Computer-Aided Design, Survival rate, Patient satisfaction, Oral Health

## Abstract

Currently, there is no consensus on the indications and clinical performance of implant-supported overdentures (IODs) involving computer-aided design and manufacturing (CAD-CAM) bars.

**Objective:**

To evaluate the performance of IODs involving CAD-CAM bars.

**Methodology:**

A comprehensive search of studies published until May 2023 was conducted in many databases, including PubMed/MEDLINE, Web of Science, Cochrane Library, and SciELO, following the Preferred Reporting Items for Systematic Reviews and Meta-Analyses (PRISMA). The population, intervention, comparison, outcome (PICO) question was: “How do IODs retained by bars fabricated by CAD-CAM technology perform in daily clinical practice?” The meta-analysis included clinical studies based on effect size and a two-tailed null test with a 95% confidence interval (CI).

**Results:**

Ten studies were included in the meta-analysis. Among them, nine reported a 100% implant survival rate for all CAD-CAM milled bars. Complications were reported in two studies with CAD/CAM-milled titanium bars, and one study reported more fractures in soldered gold bars used in maxillary rehabilitation. However, no fractures were observed in IODs retained by PEEK and zirconia bars. According to six studies, biological complications, including peri-implantitis, were minimal in the BioHPP and PEEK bar groups, while no cases were reported in the titanium or zirconia bar groups. CAD-CAM-milled zirconia bars had higher plaque and bleeding indices compared with titanium bars, as evidenced by findings from five studies. All four studies that evaluated Oral Health Impact Profile (OHIP) scores showed a positive effect of IODs retained by CAD-CAM milled titanium bars on quality of life. Patient satisfaction and acceptance by prosthodontists were significantly high, according to the results of five studies.

**Conclusion:**

Overdentures retained with CAD-CAM milled titanium bars show great potential for use in daily clinical practice. Moreover, patient and practitioner satisfaction was very high when this method was used.

## Introduction

Edentulism is a physical disability associated with tooth loss that leads to functional, aesthetic, and psychological challenges. Implant-supported overdentures (IODs) have emerged as successful treatment options, improving stability, chewing efficiency, and oral health-related quality of life. Moreover, they also preserve bone structure and offer structural benefits.^1–4^

While mandibular IODs with two interforaminal implants are considered the standard treatment, maxillary rehabilitation is more challenging and may require a minimum of four implants.^5–7^ These prostheses can be retained using various attachments, such as ball attachments, Locator^®^, Novaloc, magnetic attachments, or a bar fixed between the implants.^8–10^ However, according to Slot, et al.^11^(2010), the use of bars in overdenture rehabilitation represents the most predictable clinical approach, especially for edentulous patients in the upper jaw. This approach offers advantages, such as high levels of retention and better distribution of forces to the implants due to the splinting effect, and is the preferred option when achieving adequate implant parallelism is challenging.^12^ However, when distal extensions are used to improve the stability of the prothesis, conventional soldered extensions often fracture due to their inability to withstand the functional occlusal load.^13^

Recent advancements in computer-aided design and computer-aided manufacturing (CAD-CAM) techniques have facilitated the production of milled bars from a single block of metal or polymer, eliminating the need for soldering and other fusion processes and providing greater mechanical stability with extensions. This technology offers several advantages in terms of part quality, precision, passive fit, and biological acceptance.^14–17^

Considering the importance of improving the treatment of edentulous patients and incorporating new technologies to obtain superior devices, this systematic review and meta-analysis evaluated the clinical performance of implant-supported overdentures retained with bars fabricated using CAD-CAM systems. The null hypothesis proposed that the use of CAD-CAM bars does not improve the clinical performance of IODs.

## Methodology

### Registration, protocol, and eligibility criteria

This systematic review was registered in the International Prospective Register of Systematic Reviews (PROSPERO) under registration no. CRD 42021284190, and details of the registration can be accessed online. The study followed the guidelines and standards of the Preferred Reporting Items for Systematic Reviews and Meta-analyses (PRISMA) and the Cochrane Collaboration (Cochrane Handbook for Systematic Reviews of Interventions, version 6.2), which provide recommendations for the development of systematic reviews and meta-analysis.^18–21^

The study eligibility criteria included clinical studies that evaluated the performance of implant overdentures retained by bars fabricated using CAD-CAM methods. The following inclusion criteria were applied: studies published in English, Spanish, or Portuguese; clinical studies including randomized controlled trials, prospective clinical trials, and retrospective studies; IOD bars fabricated using CAD-CAM methods, such as printing or milling techniques; a follow-up period of less than 60 days; and studies published up to May 2023. Articles in which group data were not individualized; clinical case reports; studies with incomplete data, patients rehabilitated with mini-implants, or implant-supported overdenture bars that were not milled or printed using CAD-CAM technology; *in vitro* studies; and systematic reviews were excluded.

Moreover, this systematic review was conducted following the population, intervention, comparison, outcome (PICO) strategy, with the following question serving as the guiding framework: “How do IODs retained by bars fabricated by CAD-CAM technology perform in daily clinical practice?” The analysis was based on the following PICO index: the population of interest was patients with edentulism treated with IODs; the intervention involved IODs retained by bars fabricated using CAD-CAM technology (printing or milling techniques); the comparison groups included patients with IODs fabricated using conventional methods and patients with CAD-CAM prostheses, with baseline data available for comparison; outcomes were quantitative survival rates, marginal bone loss, complications (biological and prosthodontic), Oral Health Impact Profile (OHIP) scores, plaque and bleeding indices, and patient and practitioner satisfaction with IODs retained by CAD-CAM bars.

### Information source, search strategy, and data collection

Many databases, including MEDLINE, PubMed, Cochrane Library, SciELO, and Web of Science, were used to search for articles published until May 2023. The search strategy used MeSH/PubMed-based Boolean operators, such as “Overdentures,” “CAD-CAM BAR,” and “Digital BAR.” Supplementary Table 1 provides detailed information on the related searches conducted in these databases. Moreover, specific journals and relevant studies in the areas of IODs and digital technology were manually searched. The article selection and data collection processes were conducted by two previously trained examiners (M. D. P. and A. P. C. C.). In cases of disagreement, three examiners (J. F. S. Jr., H.S.V., and R. M. B. C.) provided clarification and resolved any discrepancies. During article selection, titles and abstracts were screened based on predefined inclusion and exclusion criteria, with a kappa test (k=1.0) applied to minimize bias.

Moreover, two examiners (V. C. P. and T. O. O. M.) provided further clarification and technical support to address any questions that arose. A consensus meeting involving all authors was held to assess the data collection process, the selected articles, and the risk of bias.

The included clinical studies were evaluated based on their methodologies and categorized into randomized controlled trials, prospective clinical trials, and retrospective studies.^22^ The data in the tables were extracted by two examiners (A. P. C. C. and H. S. V.) and cross-checked by another researcher (J. F. S. Jr.).

### Summary measures

Quantitative data were grouped according to the following variables: number of implant overdenture bar complications and biological complications divided according to the CAD-CAM and control groups. The number of IODs retained by CAD-CAM milled titanium bars and conventional IODs was considered for data analysis (dichotomous data), which was used as the risk ratio (RR), and comparisons were made between the types of overdentures.^19,20^ Statistical significance was set at p<0.05.^19–21^ The prevalence of mechanical/technical complications and biological complications was also analyzed in the CAD-CAM milled titanium bar groups. To analyze marginal bone loss in the CAD-CAM group, the mean rate of bone loss, standard deviation, and total number of implants installed were considered. This information was evaluated for the event rate, considering a 95% confidence interval (CI). The contributions of each study were assessed. Comprehensive Meta-Analysis software (version 3.0; Biostat, Englewood, NJ, USA) was used to create forest plots.^22^

Regarding the risk of bias, heterogeneity was assessed using the Q method and the I^2^ value was analyzed.^23,24^ This study adopted random analysis for all meta-analyses to reduce the potential for heterogeneity.^18^

### Data items

The collected data included the following information: (1) authors and year of publication; (2) study design; (3) evaluated data; (4) sample size; (5) materials; (6) rehabilitated arches; (7) CAD-CAM method used; (8) dental implant-supported overdentures; (9) control groups or baseline data used for comparison; and (10) follow-up duration.

### Quality assessment

The methodological quality of the selected studies was assessed using the Newcastle-Ottawa Scale (NOS), which was specifically designed to assess the quality of nonrandomized studies and consists of eight items grouped into three major components: selection, comparability, and outcome (for cohort studies) or exposure (case-control studies). A star rating system was used to perform a semi-quantitative quality assessment of each selected study.^25^ Moreover, the ROBINS-I tool was used to assess the risk of bias for nonrandomized studies. This tool was developed by members of the Cochrane Bias Methods Group and measures bias due to confounding factors, selection of participants, classification of interventions, deviations from intended interventions, missing data, measurement of outcomes, and selection of the reported result. For randomized controlled trials (RCTs), the risk of bias in randomized trials tool was applied, which assesses bias related to the randomization process, deviations from intended interventions, outcome data, outcome measurement, and selection of the reported results. Responses for the seven areas presented in the ROBINS-I tool and the five domains presented in the ROB scale were prepared using the Robvis website.^26^ The Grading of Recommendations Assessment, Development, and Evaluation (GRADE) approach was used to assess the quality of evidence for each outcome across studies. GRADE considers study design, inconsistency, indirectness, imprecision, and publication bias to assess the quality of evidence. Ratings according to GRADE are categorized into high, moderate, low, and very low. These ratings are applied to a body of evidence on the outcome assessed, but not to individual studies. The findings were compiled and summarized using the GRADEpro Guidance Development Tool (www.gradepro.org).^27^ Quality assessment was performed independently by two authors (A. P. C. C. and J. F. S. Jr.) following the established guidelines.^26,27^

## Results

**Figure 1:**
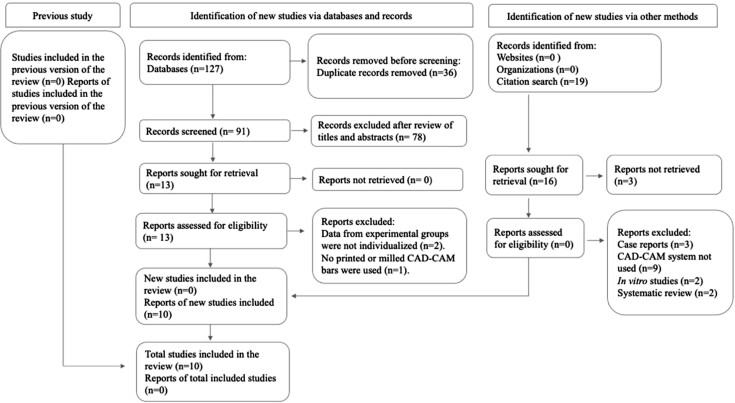
Article selection data according to the PRISMA diagram. PRISMA: Preferred Reporting Items for Systematic Reviews and Meta-Analysis

The initial search of the databases resulted in 127 references. After removing duplicates, 91 articles remained. After a thorough review of titles and abstracts, 13 articles were selected for full-text reading. Among them, three articles were excluded because they did not meet the inclusion criteria (data of experimental groups were not individualized and no printed or milled CAD-CAM bars were used). Consequently, ten studies were included in this systematic review.^14,17,28–35^ A flowchart depicting the search and selection process according to the PRISMA criteria is presented in Figure 1.

### Description of the studies

Among the ten selected articles, nine specifically examined various types of bars, including CAD-CAM milled titanium bars, BioHPP bars, PEEK bars, and zirconia bars. All studies reported 100% implant survival rates in the evaluated groups.^14,17,28–34^

Moreover, eight studies assessed prosthesis survival rates.^14,17,28–31,34,35^ The results showed no complications in the milled titanium bars or prosthesis bases after one to two years of follow-up.^28,29,31^ Katsoulis, Brunner, and Mericske-Stern^30^ (2011) reported a maxillary prosthesis fracture in the CAD-CAM milled titanium bars group; however, when comparing them with conventional bars, they found higher complication rates after two years of follow-up. Katsoulis, et al.^14^ (2015) reported no significant differences in prosthesis survival rates between CAD-CAM milled titanium bars and conventional bars after three to four years of follow-up in the mandible. These findings suggest that maxillary overdentures have a lower incidence of complications compared with mandibular overdentures. Moreover, CAD-CAM-milled titanium bars had the lowest complication rates. However, during mandibular rehabilitation, no significant differences were observed between these prostheses after a follow-up of three to four years.^14,30^

Zuercher, et al.^17^ (2022) conducted a prospective study on CAD-CAM zirconia bars and reported no fractures in the prosthesis or the bar after one year of follow-up. Mangano, et al.^34^ (2019) reported no fractures in PEEK bars after one year of follow-up. However, they observed complications related to bar adaptation, probably due to intraoral scanning issues. Al-Asad, et al.^33^ (2023) found prosthesis fractures in two implant overdentures, but no significant differences compared with hybrid prostheses. These results are detailed in Table 1.

**Table 1:** Summary of implants and prosthetic survival rates

Author	Type of study	Period of evaluation	Arch/ Patients	Groups	Number of implants	Implant Survival Rate	Number of prothesis	Bar fracture/Prosthesis Fracture	Results
Katsoulis, et al.^14^ (2015)	Retrospective	3-4 years	Mandible/213	CAD-CAM milled titanium bar	231	100%	101	9/4	Activation of matrices was required 2.4x less often in Ti-bar. No difference was observed for denture repair (fracture of base or fracture teeth).
				Soldered gold bars	246		112	9/4	
Katsoulis, Brunner and Mericske-Stern^30^ (2011)	Prospective	2 years	Maxilla/41	CAD-CAM milled titanium bar	51	100%	12	0/1	Data revealed more bar complications in the conventional group and more prosthesis failure in implant fixed prosthesis. Additionally, 11 teeth fractures were reported for conventional group, 8 for implant fixed prosthesis and 0 for IOD.
				Soldered gold bars	68	100%	16	4/1	
				Implant fixed protheses	74	100%	13	0/6	
Toia, et al.^31^ (2019)	Prospective	2 years	Maxilla/15	CAD-CAM milled titanium bar	185	100%	40	0/0	In one patient, two prosthetic screws lost their tightness in the mesostructured.
			Mandible/25						One patient reported a technical complication consisting of a chipping of the resin central lower left incisor.
Pozzi, Tallarico and Moy^28^ (2016)	Prospective	1 year	Maxilla/9 Mandible/9	CAD-CAM milled titanium bar	72	100%	18	0/0	No technical complication occurring during the follow-up, resulting in a prosthetic success rate.
Mangano, et al.^34^ (2019)	Prospective	1 year	Maxilla/15	PEEK	60	100%	15	0/0	Three out of 15 PEEK bars did not present a sufficient passive fit or adaptation. Otherwise, two overdentures need to be repaired by tooth fractures.
Cordaro, et al.^29^ (2013)	Retrospective	13 months	Mandible/19	Locator^®^	76	100%	NR	0/0	The bar group required additional visits to verify the passive fit of the bar. Furthermore, four patients in the Locator^®^ group reported experiencing soft tissue discomfort, while one patient in the bar group required a reline of the prosthesis.
		18 months	Mandible/20	CAD-CAM milled titanium bar	80	100%	NR	0/0	
Srinivasan, et al.^32^ (2020)	Prospective	1 year	Mandible/38	CAD-CAM milled titanium bar with distal extension + gold clip	19	100%	19	NR	No complications related to the prosthesis were reported in this study.
				Retentive anchors + gold matrices	19	100%	19	NR	
Zuercher, et al^.17^ (2022)	Prospective	1 year	Mandible/15	CAD-CAM zirconia bars	30	100%	15	0/0	Five patients reported sore spots and three patients showed superficial brown discoloration of the zirconia bar at the margin on the lingual side. No requirements for prosthodontic maintenance were observed.
Al-Asad, et al.^33^ (2023)	Prospective	18 months	Mandible/20	BioHPP bars	80	100%	10	NR/2	Insignificant differences (p=0.531) were found in prosthesis fracture. Additionally, the hybrid prosthesis group had two fractured crowns and the IOD group none.
				BioHPP fixed hybrid prosthesis			10	NR/1	

CAD-CAM, Computer aided design-Computer aided manufacturing; PEEK, Polyether-ether-ketone; NR, No report; IOD, implant overdenture; BioHpp, High-Performance Polymer (based on the PEEK).

**Figure 2:**
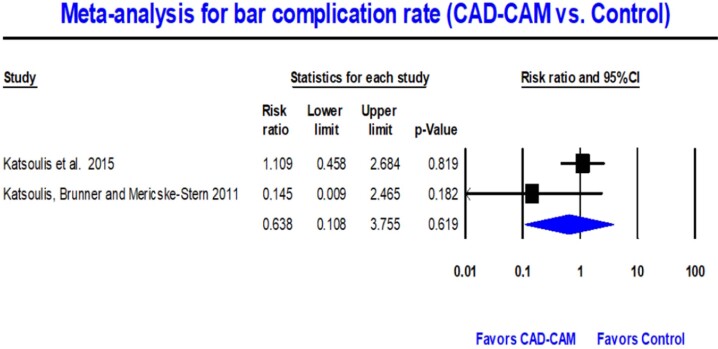
Forest plot for CAD-CAM milled titanium bar vs. control

Two meta-analyses were conducted to evaluate the complications associated with IODs retained using CAD-CAM-milled titanium bars. The first meta-analysis compared the incidence of complications between CAD-CAM milled titanium bars (n=113) and conventional gold bars (n=128) for IODs. Nine complications were identified in the CAD-CAM group, while the conventional group had 13 complications.^14,30^ The meta-analysis showed no significant difference in complications between the two methods (RR=0.638; 95% CI 0.108–3.755; p=0.619). A mean accuracy fit of 0.638 suggested a lower probability of complications in the CAD-CAM milled titanium bar group (Figure 2). Heterogeneity assessment showed homogeneity between studies, with a Q-value of 1.804 (p=0.179) and 44.561 I^2^ value (Figure 2).

**Figure 3:**
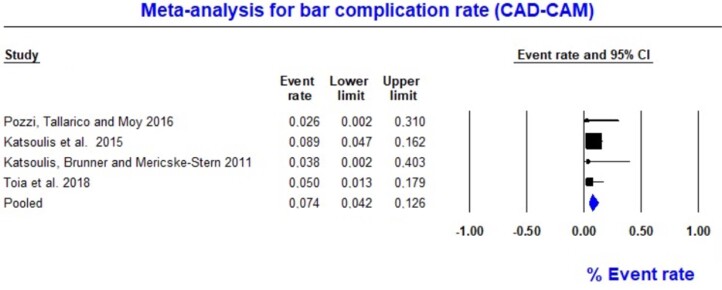
Forest plot for titanium bar complication rate (CAD-CAM)

Moreover, the event rate of complications in IODs retained by CAD-CAM-milled titanium bars was also evaluated. Four studies involving 171 IODs retained using the CAD-CAM method identified 11 complications at the bar level.^14,28,30,31^ Event rate data ranged from 4.2% to 12.6%. The overall pooled event rate was 7.4% (random; 95% CI 4.2–12.6) (Figure 3). The failure rate heterogeneity analysis yielded a Q-value of 1469 (p=0.689) and an I^2^ value of 0.0, showing homogeneity between studies (Figure 3). These analyses supported the descriptive results, suggesting a low fracture probability in CAD-CAM milled titanium bars.

Six studies were included to evaluate biological complications. Among them, two studies specifically evaluated BioHPP and PEEK bars and reported the occurrence of peri-implantitis as a complication.^33,34^ Moreover, studies evaluating titanium and zirconia CAD-CAM bars showed fewer biological complications after four and one year of follow-up, respectively.^14,17,28,31^ These findings suggest that polymer-based bars, such as BioHPP and PEEK, may be associated with unfavorable biological conditions, including peri-implantitis, after 18 months of follow-up.^33,34^ A summary of these results is presented in Table 2.

**Table 2:** Summary of biological complication

Author	Period of evaluation	Arch/Patients	Number of prosthesis/implants	Groups	Results
Katsoulis, et al.^14^ (2015)	3-4 years	Mandible/213	101/231	CAD-CAM milled titanium bars	The soft tissue hyperplasia was a common finding with gold bars (7 events), however the group of CAD-CAM milled bars exhibited 3 events.
112/246	Soldered gold bars
Pozzi, Tallarico and Moy^28^ (2016)	1 year	Maxilla/9	18/72	CAD-CAM milled titanium bar	No biologic complications occurred during the follow-up, resulting in the success of implants and prosthesis success.
Mandible/9
Toia, et al.^31^ (2019)	2 years	Maxilla/15	40/185	CAD-CAM milled titanium bars	Only one implant of 185 presented a peri-implant mucositis.
Mandible/25
Mangano, et al.^34^ (2019)	1 year	Maxilla/15	15/60	Polyether-ether-ketone (PEEK)	Two fixtures with peri-implantitis were founded in one patient.
Zuercher, et al.^17^ (2022)	1 year	Mandible/15	15/30	CAD-CAM zirconia bars	One patient showed generalized mucosal hyperplasia around the zirconia bar.
Al-Asad, et al.^33^ (2023)	18 months	Mandible/20	10/40	BioHPP bars	Three patients of the hybrid group and two patients of the bars group presented periimplantits. In addition, three patients presented Hyperplasia under frameworks and two under bars.
			10/40	BioHPP fixed hybrid prosthesis	

CAD-CAM, Computer aided design-Computer aided manufacturing; PEEK, Polyether-ether-ketone; NR, No report; BioHpp, High-Performance Polymer (based on the PEEK).

**Figure 4:**
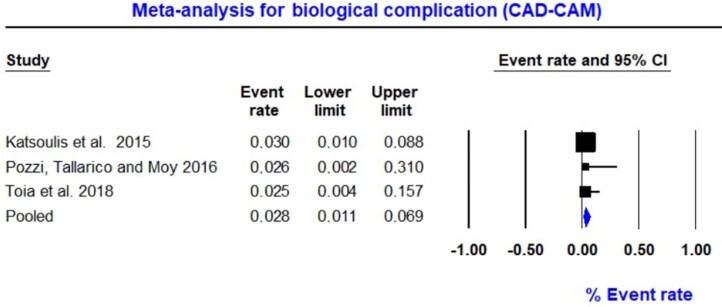
Forest plot for biological complication rate (CAD-CAM milled titanium bars)

The rate of biological complications in the CAD-CAM group was assessed using a meta-analysis. In three studies involving 159 IODs retained by CAD-CAM-milled titanium bars, four biological complications were identified.^14,28,31^ Event rate data ranged from 1.1% to 6.9%. The overall pooled event rate was 2.8% (random; 95% CI 1.1–6.9), showing a low probability of biological complications in CAD-CAM milled titanium bars. The heterogeneity of the failure rate was considered as the Q-value (Q=0.026; p=0.987; I^2^=0.0), highlighting the homogeneity of all studies involved in this analysis (Figure 4).

**Figure 5:**
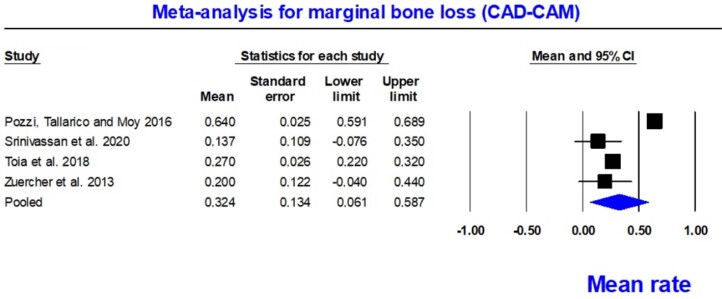
Forest plot for marginal bone loss rate (CAD-CAM)

Peri-implant marginal bone loss was an additional aspect evaluated in this study, including four of the selected studies.^17,28,31,32^ Both zirconia and titanium bars had similar bone loss, suggesting that the presence of bone loss in rehabilitation treatment does not depend on the type of metal used for bar fabrication. Moreover, titanium bars showed values comparable to those of bars fabricated using conventional techniques. Detailed data are shown in Table 3. For the meta-analysis of peri-implant marginal bone loss, four studies involving 324 implants were included.^17,28,31,32^ These implants were used for overdentures retained with titanium or zirconia CAD-CAM bars, with follow-up periods from 12 to 24 months. The mean marginal bone loss rate ranged from 0.061 to 0.587. The overall pooled mean rate was 0.324 mm (random; 95% CI 0.061–0.587). The meta-analysis showed a high level of heterogeneity, as highlighted by the Q-value of 120.308 (p=0.0; I^2^=97.506) (Figure 5). This high heterogeneity suggested variations among the studies included in the analysis. A possible reason for this heterogeneity could be a specific subgroup within the study that had greater bone loss at baseline.^28^ Factors such as variations in surgical techniques and the learning curve of the professionals involved may have contributed to this variation.

**Table 3:** Data of peri-implant marginal bone level

Author	Period of evaluation	Groups	Number of implants	Mean + SD (mm)	P-value
Srinivassan, et al.^32^ (2020)	1 year	CAD-CAM milled titanium bar + gold clip	38	0.137 + 0.671	0.754
		Retentive anchors + gold matrices	38	0.205 + 0.64	
Pozzi, Tallarico and Moy^28^ (2016)	1 year	CAD-CAM milled titanium bar	72	0.64 + 0.21	0.003
Toia, et al.^31^ (2019)	2 years	CAD-CAM milled titanium bar	185	0.27 + 0.35	P>0.05
Zuercher, et al.^17^ (2022)	1 year	CAD-CAM zirconia bars	30	0.20 + 0.67	NR

CAD-CAM, Computer aided design-Computer aided manufacturing.

Plaque and bleeding indices were assessed in this systematic review using data from five selected studies.^17,28,29,31,32^ Among them, four studies reported favorable indices for CAD-CAM milled titanium barrehabilitations,^28,31,32^ while one study highlighted lower indices for Locator^®^ attachments.^29^ However, Zuercher, et al.^17^ (2022) stated that even better results can be expected for zirconia bars, for which no plaque accumulation or bleeding was found. Supplementary Table 2 presents further details.

Four of the selected studies assessed the effect of oral disorders on patients with implant-supported overdentures retained by CAD-CAM milled titanium bars using the Oral Health Impact Profile (OHIP) questionnaire.^28,30–32^ All studies reported a significant improvement in OHIP scores, showing that CAD-CAM-milled titanium bars substantially improved the quality of life of patients undergoing rehabilitation with these prostheses. Detailed data are provided in Supplementary Table 3.

Regarding patient and practitioner satisfaction, three studies showed positive patient satisfaction with CAD-CAM milled titanium bars.^29,31,32^ However, one study highlighted that patients undergoing rehabilitation with Locator^®^ attachments reported easier oral hygiene maintenance.^29^ Altonbary and Emera^35^ (2021) found higher satisfaction with zirconia bars in terms of appearance, comfort, and hygiene compared with conventional Co-Cr bars. Moreover, there were no significant differences in satisfaction between BioHPP bar-retained overdentures and BioHPP-fixed hybrid prostheses.^33^ Overall, CAD-CAM bars had positive outcomes in terms of patient satisfaction with IODs. Different types of bars, including titanium, zirconia, and BioHPPs, offered varying levels of patient satisfaction, ease of oral hygiene, and practitioner preferences. Supplementary Table 4 provides details.

### Quality assessment of individual studies

Assessment of bias in the selected studies showed that most had good methodological quality, especially those with control groups.^14,30,32,33,35^ Table 4 presents detailed data. However, one study scored 7 due to a lack of reporting on how the comparability of selected cases was controlled.^29^ Further details are provided in Supplementary Table 5. The cohort studies included in the review scored 7 due to inadequate follow-up duration (up to two years).^28,31,34^ A study scored 6 due to the wide age range of the sample, which may have caused significant differences in the results^17^. Supplementary Table 6 provides additional details.

**Table 4:** Quality assessment of the reviewed studies

Author	Study Design	Implants	Mean age, years	Arch/Patients	Materials/ Fabrication	Range of follow-up	Level of evidence NHMRC	NOS
Katsoulis, et al^.14^ (2015)	Retrospective	477	68 + 10 years	Mandible/112	Soldered gold bars (Dolder bar attachment macro; Cedres+Métaux AS)	3-4 years	III-2	9
				Mandible/101	Titanium (Ti6Al4V)/ milling technique (Procera, Nobel Biocare)			
Katsoulis, Brunner and Mericske-Stern^30^ (2011)	Prospective	193	63.3 ± 6.1 years	Maxilla/16	Soldered gold bars (Dolder bar)	2 years	III-2	9
				Maxilla/12	Bar IOD, Titanium/milling technique (Procera, Nobel Biocare)			
				Maxilla/13	Framework IFP, Titanium/milling technique (Procera, Nobel Biocare)			
Toia, et al.^31^ (2019)	Prospective	185	69 ± 9.5 years	Maxilla/15	Titanium/milling technique (Dentsply, Sirona).	2 years	III-2	7
Pozzi, Tallarico and Moy^28^ (2016)	Prospective	72	65.4 years	Mandible, Maxilla/18	Titanium/milling technique (Procera, Nobel Biocare).	1 year	III-2	7
Mangano, et al^.34^ (2019)	Prospective	60	68.8 ± 4.7 years	Maxilla/15	PEEK/milling technique (DWX-51^®^, Roland EasyShape)	1 year	III-2	7
Srinivassan, et al.^32^ (2020)	Randomized controlled trial	76	Control group: 74.7 ± 7.8 / Experimental group: 70.3 ± 10.7	Mandible/19	Retentive anchors + gold matrices (Dalbo^®^ PLUS, Cendres + Métaux AS)	1 year	II	9
				Mandible/19	Titanium/milling technique + gold clip (Cares^®^, Institut Straumann AG)			
Altonbary and Emera^35^ (2020)	Pseudorandomized	40	55-75 years	Mandible/10	Zirconia bar (Zenostar MO 2, Ivoclar Vivadent)/ milling technique	3 months	III-1	9
				Mandible/10	Co-Cr/ Casted technique			
Cordaro, et al.^29^ (2013)	Retrospective	156	62.1	Mandible/19	Locator^®^ (Zest Anchors LLC)	1 year	III-3	9
				Mandible/20	Titanium/milling technique CAM StructSURE^®^ (Biomet 3i)			
Zuercher, et al.^17^ (2022)	Prospective	30	18-89 years	Mandible/15	CAD-CAM zirconia bars (Metoxid, Thayngen, Switzerland)/ milling technique	1 year	III-2	7
Al-Asad, et al.^33^ (2023)	Prospective	80	50-65 years	Mandible/10	BioHPP bars/ milling technique	18 months	III-2	9
				Mandible/10	BioHPP fixed hybrid prosthesis/ milling technique			

CAD-CAM, Computer aided design-Computer aided manufacturing; PEEK, Polyether-ether-ketone; BioHpp, High-Performance Polymer (based on the PEEK).

**Figure 6:**
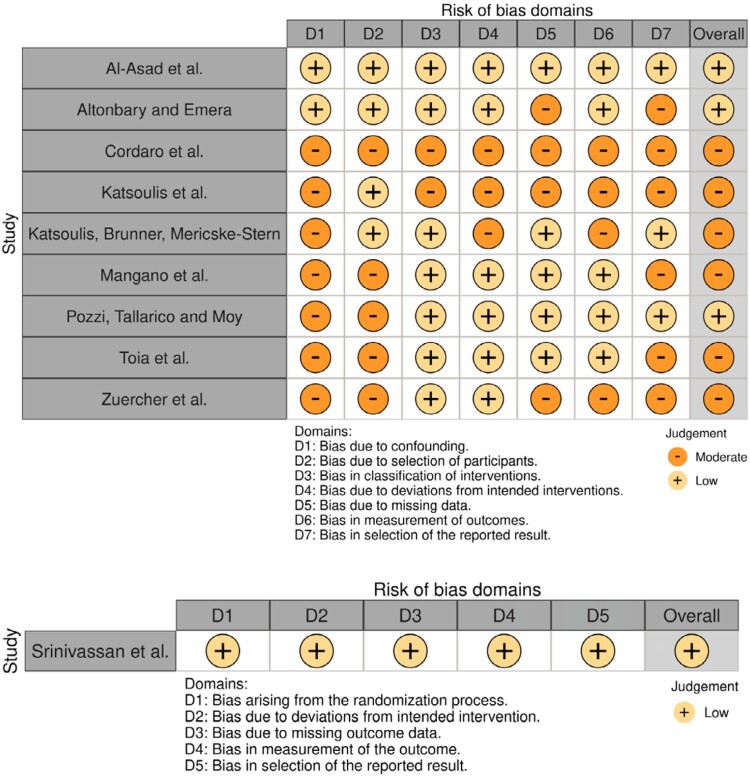
Risk of bias ROBINS-I and ROB – General information

The analysis of randomized controlled trials showed no risk of bias,^32^ while nonrandomized studies had biases in patient selection due to retrospective studies, lack of sample calculation, lack of randomization, small sample sizes, absence of control groups, deficiencies in intervention analysis, descriptive analysis of primary data, and selective dissemination of information due to short follow-up periods.^14,17,28–31,33–35^ Data from ROBINS-I and ROB are shown in Figure 6. The overall quality of evidence for the main outcomes ranged from very low to moderate, based on the GRADE approach. Study design limitations, inconsistencies, indirectness, and publication bias contributed to this assessment. The evidence for technical complications of CAD-CAM bars and marginal bone loss in CAD-CAM bars was rated as low due to several factors, such as random sequence generation, allocation concealment, different conditions, small sample sizes, and short follow-up periods. Further details are provided in Appendix 1 in the Supplementary Material.

## Discussion

The results of this systematic review and meta-analysis supported our null hypothesis that CAD-CAM milled bars used in implant-supported overdentures had excellent performance in daily clinical practice. Many outcome measures were assessed, including implant and prosthesis survival rates, biological complications, marginal bone loss, plaque and bleeding indices, OHIP scores, and patient and practitioner satisfaction.

Nine selected articles evaluating implant survival reported a 100% success rate during a follow-up period of up to four years, showing the safety of CAD-CAM milled titanium, PEEK, zirconia, and BioHPP bars.^14,17,28–34^ However, peri-implantitis occured in a short-term follow-up of polymer-based bars (BioHPP and PEEK).^33,34^ It is possible that an error in the bar design limited the space between the mucosa and the bar, significantly affecting patients’ oral hygiene maintenance. Moreover, polymer-based bars may be more susceptible to microbial accumulation. According to Wiessner, et al.^36^ (2023), there is greater microbial accumulation on polymer-based bars (PEEK and BioHPP) compared with titanium or zirconia bars. In contrast, Hahnel, et al.^37^ (2015) highlighted that microbial adhesion on PEEK bars was equal to or lower than that on zirconia and titanium bars. These findings are supported by Barkamo, et al.^38^ (2019), who found no significant differences in microbial accumulation between PEEK and titanium bars, but observed that increasing the roughness of PEEK bars significantly increased microbial adhesion. These complications should be considered and further studies should be conducted to confirm whether polymer-based bars increase surface roughness over time and are more prone to microbial adhesion. The study also assessed prothesis survival rate and the analysis suggested a low fracture probability in IODs retained by CAD-CAM milled titanium bars.^14,28–32^ However, two articles identified issues related to bar and prosthesis fractures.^14,30^ A study found fewer fractures in maxillary IODs with CAD-CAM bars compared with conventional gold bars, which may be attributed to the absence of welding in CAD-CAM bars.^30,39^ Another study found no statistically significant differences between gold and CAD-CAM bars for mandibular rehabilitation.^14^ These findings suggest that maxillary IODs have a lower incidence of complications and that both conventional and CAD-CAM techniques are effective in mandibular prosthetic rehabilitation. However, further research and long-term follow-up are required to validate these findings and gain more insight into the long-term outcomes of IODs.

In rehabilitation with zirconia or PEEK bars, no bar or prothesis fractures were reported.^17,34^ However, complications, such as insufficient passive fit occurred in the PEEK group and may be attributed to oral scanning strategy issues.^34^ Moreover, two fractures were identified in implant-supported overdentures retained by BioHPP bars.^33^ These fractures could potentially be due to the larger volume required for the bars and the compromised thickness of the subsequent overdentures. Further studies are needed to validate these observations and provide more information on these complications.

Plaque and bleeding indices are reliable indicators of oral hygiene, with exceptional outcomes for CAD-CAM-milled titanium bar rehabilitations.^28,29,31,32^ Prosthesis removability significantly enhances patient hygiene, especially in older individuals, who may face age-related motor coordination challenges.^40^ However, Cordaro, et al.^29^ (2013) reported superior rehabilitation outcomes using Locator^®^ attachments, potentially due to their smaller dimensions, which facilitated enhanced cleaning efficacy. Moreover, Zuercher, et al.^17^ (2022) showed that the absence of plaque or bleeding indices was associated with zirconia bars, highlighting their immense potential in preserving exceptional oral hygiene standards.

This study evaluated the effect of oral disorders on oral health-related quality of life (OHRQoL) of patients with implant-supported overdentures. The data collected showed a significant reduction in OHIP scores after one year of follow-up in all patients, including those rehabilitated with conventional bars.^28,30–32^ These findings are consistent with previous studies in this field.^41^ Interestingly, in the CAD-CAM-milled titanium bar group, OHIP scores substantially decreased as early as the second week of follow-up, showing a potentially faster improvement in patients rehabilitated with CAD-CAM-milled titanium bars. Moreover, Katsoulis, Brunner, and Mericske-Stern^30^ (2011) reported significantly lower OHIP scores in an implant-supported fixed prosthesis group. These findings suggest that the enhanced aesthetics, phonetics, and chewing efficiency associated with implant-fixed prostheses may have influenced the observed discrepancies in OHIP scores between the groups. Implant-supported overdentures rely on both the implants and the mucosa for support, which may lead to greater discomfort compared with implant-supported fixed prostheses.

Regarding patient and practitioner satisfaction associated with CAD-CAM milled bars in implant overdenture rehabilitation, the findings showed that patients treated with CAD-CAM milled titanium bars had significantly better overall satisfaction.^29,31,32^ However, considerations such as hygiene comfort favored the Locator^®^ group over the CAD-CAM group.^29^ Patients expressed greater satisfaction with zirconia bars, which was attributed to their aesthetics, time-saving fabrication, and improved oral hygiene maintenance.^35^ Moreover, BioHPP bars had satisfactory patient acceptance, comparable to implant-fixed prostheses, possibly due to the convenience of removing the prosthesis for hygiene maintenance.^33^ Practitioners expressed high satisfaction with CAD-CAM-milled titanium bars, although one study shows a preference for Locator^®^ attachments due to superior soft tissue conditions and hygiene maintenance.^29^

Based on the findings of this systematic review, CAD-CAM milled titanium bars are a highly favorable treatment option for completely edentulous patients in both the maxilla and mandible with a follow-up period of up to four years. However, further validation of these results requires additional long-term randomized and prospective clinical studies comparing IODs supported by CAD-CAM-milled titanium bars and conventional overdentures. Moreover, although PEEK, BioHPP, and zirconia bars have shown promising short-term performance, the evaluation of their long-term effectiveness in edentulous therapy needs further randomized and prospective studies.

This systematic review included a diverse range of prospective, retrospective, randomized controlled, and pseudorandomized trials. However, it is important to acknowledge a notable limitation of this review, which was the limited availability of short- and long-term randomized and prospective clinical studies specifically evaluating the performance of CAD-CAM milled bars and comparing them with conventional implant overdentures. Thus, conducting randomized and prospective studies that comprehensively evaluate the performance of CAD-CAM milled bars over various time periods is essential.

## Conclusion

Within the limitations of this review, we concluded that IODs retained by titanium bars designed using CAD-CAM methods have optimal performance in daily clinical practice. They showed high implant survival rates, minimal biological and mechanical complications, and a significantly improved patient quality of life. Moreover, PEEK, BioHPP, and zirconia bars seem to be promising materials for overdenture rehabilitation. However, further studies should be conducted to confirm these findings.

## Data Availability

The datasets generated and analyzed during the current study are available in the SciELO Data, https://doi.org/10.48331/scielodata.GNMP3T
